# Location awareness using combined multimodal sensor infrastructure for emergency service personnel

**DOI:** 10.1186/2046-7648-4-S1-A29

**Published:** 2015-09-14

**Authors:** Miklos Kozlovszky, Daniela Zavec Pavlinic, Gábor Fehér

**Affiliations:** 1 Biotech Knowledge Center, Obuda University, Budapest, Hungary; 2 Titera Ltd., Slovenia; 3 OMTLAB Research Ltd, Hungary

## Introduction

Location awareness is crucial to effectively work or even survival in a harsh environment. Besides basic functions of protection, the new generation of protective garments has additional functions like monitoring the environment and provides location information about highlighted objects, spots, or personnel. This is a challenging task [1]; location sensors range from low accuracy sensors with almost global coverage to high accuracy short range solutions. Design requirements such as cost, bulkiness, accuracy, independency, scalability, robustness are key parameters [2]. We are designing and developing an intelligent Personal Protective System (PPS) integrated with a data acquisition (DAQ) system and we are using enabling technologies to collect location information and a wide range of environment information. In this paper we show how our first prototype of location monitoring is designed and developed.

## Methods

The proposed localization solution is based on a combination of different technologies. Most of the technologies are using radio wave communication. We are tracking the position of the PPSs, combining absolute and relative positioning techniques. Relative positioning is measuring signal strength difference; thus works with relative distance and direction of arrival measurements. Absolute measurements are done when a signal from known position fixed elements can be detected. Fixed elements are the satellites emitting GPS signals and fire-engines at known locations providing ranging functions in the UHF band. We acquire periodically from the different sensor devices location information.

## Results

For outdoor environments, if GPS satellite signals and GSM signals are available, we mostly rely on this information (here the range is large, but accuracy is poor). In indoor environments the distance measurements are more suitable, and positions are estimated from triangulations among the different signal senders. The distance measurements are done in parallel with UHF and UWB radio technology. UHF distance measurements provides below 5 m accuracy in a long range (200 m-1500 m depending on environment) while UWB distance measurements give an excellent 1 m accuracy in a shorter range (20 m-100 m depending on environment). For offline movement tracking and for the support of position estimation in extreme conditions we acquire also Inertial Measurement Unit (IMU) information, which provides multi-axis acceleration information. To do location sensor data fusion in an effective way, we introduced a common representation format for the acquired sensor data. We are using a primary fusion algorithm to convert the received location information and construct a common coordinate system. The different technologies provides different operational parameters (such as availability, accuracy, etc.), which are related directly to the surrounding PPS environment. We are also evaluating the correctness of the received location data, because extreme ever-changing conditions can bring weird sensing situations.

## Conclusion

We have designed and realized a location aware solution using combined multi-modal sensor infrastructure for emergency service personnel. During location sensor fusion we convert the data to a common representation format and have created a common coordinate system. The acquired data is processed on different levels, and we evaluate/filter sensor data in real-time. The combination of the sensors can provide better location awareness even in special locations/ extreme environments for the PPS users. The acquired location data can be visualized by the PPS user, and can be transmitted towards a remote location for visualization of the emergency service team activities.
